# Effects of an Herbal Formulation Ethanolic Extract on *Streptococcus pyogenes*: Bactericidal Activity, Biofilm Control, and Interactions with Conventional Antibiotics

**DOI:** 10.3390/pathogens15090985

**Published:** 2026-09-16

**Authors:** Rohana Dolee, Oraphan Sakulkeo, Suttiwan Wunnoo, Auemphon Mordmuang, Katesarin Maneenoon, Waranya Thipkham, Supayang Piyawan Voravuthikunchai, Friedrich Götz, Arif Luqman, Jongkon Saising

**Affiliations:** 1 School of Health Science, Mae Fah Luang University, Muang Chiang Rai 57100, Chiang Rai, Thailand; 6451811005@lamduan.mfu.ac.th (R.D.); waranyath.tk@gmail.com (W.T.); 2 Biomedical Technology Research Group for Vulnerable Populations, Mae Fah Luang University, Muang Chiang Rai 57100, Chiang Rai, Thailand; 3 Traditional Thai Medical Research and Innovation Center, Faculty of Traditional Thai Medicine, Prince of Songkla University, Hat Yai 90110, Songkhla, Thailand; oraphan.s@psu.ac.th (O.S.); katesarin.m@psu.ac.th (K.M.); 4 Center of Antimicrobial Biomaterial Innovation-Southeast Asia, Faculty of Science, Prince of Songkla University, Hat Yai 90110, Songkhla, Thailand; suttiwan.w@psu.ac.th (S.W.); supayang.v@psu.ac.th (S.P.V.); 5 Division of Biological Science, Faculty of Science, Prince of Songkla University, Hat Yai 90110, Songkhla, Thailand; 6 Department of Medical Sciences, School of Medicine, Walailak University, Tha Sala 80160, Nakhon Si Thammarat, Thailand; auemphon.mo@wu.ac.th; 7 Microbial Genetics, Institute of Microbiology and Infection Medicine Tübingen, University of Tübingen, 72076 Tübingen, Germany; friedrich.goetz@uni-tuebingen.de; 8 Biology Department, Institut Teknologi Sepuluh Nopember, Surabaya 60111, Indonesia; arif.luqman@its.ac.id

**Keywords:** *Streptococcus pyogenes*, antibacterial activity, herbal formulation, antibiotic combination, antibiofilm

## Abstract

*Streptococcus pyogenes* is a pathogen that causes skin infections worldwide. Currently, there is growing interest in herbal formulations as potential sources of antibacterial agents. This in vitro study investigated the antibacterial and antibiofilm activities of herbal formulation ethanolic extract (HFE), alone and combined with specific antibiotics, against *S. pyogenes*. The minimum inhibitory concentration (MIC) and minimum bactericidal concentration (MBC) of HFE were determined using the broth microdilution method. Bactericidal activity was further evaluated using time-kill assays. Bacterial cell morphology was examined using electron microscopy. The interaction between HFE and specific antibiotics was assessed using the checkerboard assay. The MIC and MBC values of HFE against *S. pyogenes* ATCC 19615 were 16 and 32 µg/mL, respectively, while all clinical isolates showed MIC and MBC values of 8 µg/mL. Time-kill curve analysis demonstrated HFE’s bactericidal activity within 2 h and induced ultrastructural changes in *S. pyogenes* cells. HFE also exhibited antibiofilm activity against both biofilm formation and established biofilms. The checkerboard assay showed indifferent interactions, with a fractional inhibitory concentration (FIC) index of 0.57–3.00 for all HFE combinations against *S. pyogenes*. These findings suggest the potential of HFE as a natural health product for the management of *S. pyogenes* infections.

## 1. Introduction

One of the most significant bacterial causes of global skin and soft tissue infections (SSTIs) is *Streptococcus pyogenes*, commonly known as group A streptococcus (GAS), a Gram-positive bacterium [1]. SSTIs range from uncomplicated cases to potentially lethal necrotizing fasciitis [2]. This bacterium is frequently responsible for bacterial pharyngitis, impetigo, cellulitis, erysipelas, myonecrosis, and streptococcal toxic shock syndrome (StrepTSS) [3,4]. Untreated *S. pyogenes* infections can progress to serious suppurative infections or non-suppurative consequences, including rheumatic heart disease [5]. Furthermore, biofilm is one of the most significant virulence properties contributing to the severity and dissemination of *S. pyogenes* infections, affecting morbidity and mortality [6]. *S. pyogenes* isolated from muscle and soft tissue from necrotizing soft tissue infection (NSTI) patients form biofilms [7]. Biofilms allow bacteria to evade host immunity and protect against antibiotic treatment [8]. Therefore, different approaches are required to combat biofilm-related streptococcal infections.

Medicinal plants become an effective alternative, especially in developing countries with resource limitations, where natural substances hold greater potential [9]. Additionally, due to increasing bacterial resistance and adverse drug events in the current situation [10,11,12,13,14], drug discovery remains an essential priority to address issues related to antibiotic-resistant organisms [15]. This issue emphasizes the need to explore approaches to eradicate infections, particularly those resistant to antibiotics [16]. In Thailand, folk healers represent valuable local wisdom for treating various diseases, including skin diseases, and they employ herbal formulations in their approaches. Previous studies have demonstrated the antibacterial activity of herbal formulations against a variety of pathogenic bacteria, including *S. pyogenes* [17,18,19]. In addition, researchers have increasingly explored the combination of traditional medicines with antibiotics, which have shown increased efficacy against bacteria. The results of combination therapies have been remarkable; for example, Triphala, an indigenous medical product, is used for various diseases, such as skin wounds, tumors, upper respiratory diseases, and ulcers. It exhibited synergistic activity with gentamicin against selected multidrug-resistant (MDR) Gram-negative bacilli and with oxacillin against methicillin-resistant *Staphylococcus aureus* (MRSA) isolates [20]. These findings support further investigation of traditional herbal formulations as potential sources of antimicrobial agents and their interactions with conventional antibiotics.

Our previous ethnopharmacological study highlighted the important role of traditional healers in the treatment of skin diseases using herbal formulations prepared from a variety of medicinal plants and different traditional methods of preparation. The medicinal plants included in the formulation were selected based on traditional wisdom, empirical knowledge, and long-term experience [21]. However, scientific evidence supporting the biological activities of these herbal formulations remains limited. In addition, traditional preparation methods may not be suitable for standardized laboratory preparation. Therefore, the present study employed an ethanolic extract of a traditional herbal formulation to evaluate its antibacterial activity against *S. pyogenes*. The formulation, used by a local healer in Songkhla Province, Thailand, for the treatment of skin diseases, consists of three medicinal plants: *Anacardium occidentale* L.; *Zingiber montanum* (J. Koenig) Link ex Dietr.; and *Aloe vera* (L.) Burm. f. Previous studies have reported that these medicinal plants possess antibacterial activity against Gram-positive bacteria, including *S. pyogenes* and *S. aureus* [22,23,24]. The present study aimed to evaluate the antibacterial and antibiofilm activities of the ethanolic extract of this herbal formulation against *S. pyogenes* and to assess its interaction with conventional antibiotics commonly used for the treatment of skin infections.

## 2. Materials and Methods

### 2.1. Microorganisms and Culture Conditions

*S. pyogenes* ATCC 19615 was used as the reference strain. Ten clinical isolates of *S. pyogenes* were obtained from Assoc. Prof. Dr. Auemphon Mordmuang, School of Medicine, Walailak University, Nakhon Si Thammarat, Thailand. The archived clinical isolates were originally obtained from Maharaj Nakhon Si Thammarat Hospital, where they had been recovered and identified by the hospital microbiology laboratory as part of routine clinical diagnostic services. No additional patient specimens were collected specifically for this study, and no patient-identifiable information was accessed. The bacterial isolates were transferred to and handled at Walailak University in accordance with the institutional biosafety regulations and standard operating procedures. Since this study involved only previously archived, de-identified bacterial isolates and did not involve human participants or identifiable patient data, it was exempt from ethical review by the Mae Fah Luang University Ethics Committee on Human Research (Certificate of Exemption No. COE 265/2021). All bacterial strains were maintained in brain heart infusion broth (BHIB; Difco, Sparks, MD, USA) supplemented with 20% glycerol at −80 °C and cultured on brain heart infusion agar (BHIA) at 37 °C for 18–24 h before use.

### 2.2. Antibiotics

The antibiotics used in this study were amoxicillin, cephalexin, and mupirocin procured from HiMedia, Mumbai, India. Trimethoprim, clindamycin, and vancomycin were obtained from Gold Biotechnology, St. Louis, MO, USA, while ampicillin, erythromycin, and fusidic acid were sourced from Sigma-Aldrich, St. Louis, MO, USA. These antibiotics are commonly employed for the treatment of skin infections.

### 2.3. Preparation of the Herbal Formulation Ethanolic Extract

The herbal formulation consisted of the pericarps of *Anacardium occidentale* L., rhizomes of *Zingiber montanum* (J. Koenig) Link ex Dietr., and dried leaf latex of *Aloe vera* (L.) Burm. f. The traditional knowledge of this herbal formulation was provided by Mr. Somporn Chanwanisakul, a traditional practitioner at the Faculty of Traditional Thai Medicine, Prince of Songkla University, Thailand [21]. *A. occidentale* pericarps and *Z. montanum* rhizomes were collected from Chiang Mai and Surat Thani provinces, Thailand, respectively. Dried *A. vera* leaf latex was purchased from a Thai herbal store in Hat Yai, Songkhla Province. The plant materials were authenticated by Assistant Professor Dr. Katesarin Maneenoon, an ethnobotanist at the Faculty of Traditional Thai Medicine, Prince of Songkla University. Voucher specimens were deposited at the Faculty of Traditional Thai Medicine, Prince of Songkla University, Songkhla, Thailand. The voucher numbers were NK 080 for *A. occidentale*, NK 125 for *Z. montanum*, and NK153-CD for dried *A. vera* leaf latex. Quality control of *Z. montanum* rhizomes and *A. vera* dried leaf latex was performed according to the Thai Herbal Pharmacopoeia (THP) 2021 [25] and the Thai Pharmacopoeia II, Supplement 2024 [26], respectively. As no official monograph is available for *A. occidentale*, the quality assessment of its pericarps was performed using adapted pharmacopoeial methods. The plant materials were weighed in a 1:1:1 ratio, dried, and ground into a powder. The combined powder was macerated in 95% ethanol at room temperature for seven days with periodic agitation. The ethanolic extract was subsequently filtered, concentrated using a rotary vacuum evaporator (60 °C), and stored at 4 °C until use. The dried extract was dissolved in dimethyl sulfoxide (DMSO) before use.

### 2.4. Determination of the Minimum Inhibitory Concentration (MIC) and Minimum Bactericidal Concentration (MBC)

The MIC of the ethanolic extract of the herbal formulation and antibiotics was determined using the broth microdilution method following the Clinical and Laboratory Standards Institute [27]. Briefly, the ethanolic extract and antibiotics were serially two-fold diluted in a sterile microtiter plate. Bacterial strains were cultured on BHIA for 24 h, and log-phase culture was prepared in BHIB (Difco, MI, USA). The bacterial turbidity at 0.5 McFarland was achieved in a 0.85% normal saline solution (NSS) and further diluted to 1 × 10^6^ CFU/mL and then was added into 96-well plate, followed by incubation at 37 °C for 16–18 h. Turbidity of the medium indicated bacterial growth. Accordingly, 1% DMSO served as a negative control for ethanolic extract-treated cells, while MHIB was a negative control for antibiotic-treated cells, and the experiment was conducted in triplicate. Wells without visible turbidity (MIC and higher dilutions) were further streaked onto BHIA plates. After incubation at 37 °C for 24 h, the minimum concentration with no growth of tested organisms was considered the MBC value.

### 2.5. Time-Kill Study

The time-kill assay of the extract on *S. pyogenes* ATCC 19615 was performed using the viable cell count. A bacterial suspension at 1 × 10^6^ CFU/mL was added to BHIB supplemented with the ethanolic extract at different concentrations and incubated at 37 °C. Mupirocin, an antibiotic commonly used as a topical antibiotic for skin infections, was included in this experiment. At the designed time intervals, each bacterial suspension was sampled, and visible colonies were determined using the drop plate method. Time-kill curves were plotted as log colony number (CFU/mL) over time. The experiment was conducted in duplicate, and data are presented as means with ranges.

### 2.6. Bacterial Cell Morphological Observation by Scanning Electron Microscopy

To examine the bacterial cell morphology after treatment with HFE, *S. pyogenes* ATCC 19615 (10^8^ CFU/mL) was treated with the ethanolic extract at 2 × MIC and incubated at 37 °C for 24 h to obtain sufficient bacterial cells for SEM analysis. The samples were washed three times with PBS, fixed in 2% glutaraldehyde (Electron Microscopy Sciences, Hatfield, PA, USA) in PBS for 2 h, and washed with PBS. After washing with distilled water, the specimens were dehydrated in a graded ethanol series (50–100%), mounted on aluminum stubs, allowed to dry, and then coated with gold. The samples were examined under a scanning electron microscope (Quanta 400 FEG; FEI, Hillsboro, OR, USA).

### 2.7. Bacterial Cell Structure Observation by Transmission Electron Microscope (TEM)

*S. pyogenes* ATCC 19615 (10^8^ CFU/mL) was treated with the extract at 2 × MIC and incubated at 37 °C for 24 h to obtain sufficient bacterial cells for TEM analysis. The bacterial cells were then centrifuged at 5000 rpm for 5 min. After fixation with 2.5% glutaraldehyde in PBS, the cells were dehydrated in an ethanol series. The samples were embedded in Quetol resin, sectioned using an ultramicrotome, and observed under a TEM (JEOL JEM-2010, JEOL Ltd., Tokyo, Japan).

### 2.8. Effects of the Herbal Formulation Ethanolic Extract on S. pyogenes’ Biofilm Formation

The effect of HFE on *S. pyogenes* biofilm formation was determined according to the protocol previously described [28]. Briefly, *S. pyogenes* culture was incubated in TSB supplemented with sub-inhibitory concentrations of the ethanolic extract in a polystyrene 96-well plate at 37 °C for 24 h. Bacterial inoculum with 1% DMSO under the same conditions was used as the negative control. After incubation, the free-floating cells were removed, and the wells were gently washed twice with phosphate-buffered saline (PBS). The plates were air-dried, and the biofilms were then stained with 0.1% crystal violet for 30 min. The dye solution was removed, and the wells were then washed with distilled water and left to dry. Next, DMSO was added to each well, and the absorbance was measured at 570 nm.

### 2.9. Effects of the Herbal Formulation Ethanolic Extract on S. pyogenes’ Established Biofilms

MTT assay was used to evaluate the effect of the ethanolic extract on *S. pyogenes* established biofilms as previously described [28]. Briefly, bacterial inoculum in a 96-well plate was incubated at 37 °C for 24 h. The suspension was discarded, and PBS was used to remove the unattached cells. TSB containing different concentrations of HFE was added to the wells. After incubation at 37 °C for 24 h, the culture medium was replaced with MTT solution, and the plate was then incubated for 2 h. The suspension was discarded, and DMSO was added to the well, followed by absorbance measurements at 570 nm.

### 2.10. Observation of S. pyogenes Biofilms Using Scanning Electron Microscope (SEM)

To observe biofilm formation, *S. pyogenes* was cultured in a 12-well plate containing sterile glass slides and TSB—supplemented with the polyherbal ethanol extract at 1/4 × MIC—and incubated at 37 °C for 24 h. For established biofilms, bacterial culture was grown on a sterile glass slide at the bottom of a well plate for 24 h to form biofilms. The slides were washed twice with PBS and transferred to a 12-well plate containing 8 × MIC of the ethanol extract in TSB and incubated at 37 °C for 24 h. The samples were prepared as described in Section 2.6 and examined using SEM (Tescan MIRA, TESCAN, Brno, Czech Republic).

### 2.11. Combination Effects of the Herbal Formulation Ethanolic Extract and Antibiotics

The combined effect of HFE and antibiotics was evaluated using the checkerboard assay in 96-well microtiter plates according to previous studies with slight modifications [29,30]. A log-phase bacterial suspension was prepared in BHIB and diluted to 1 × 10^6^ CFU/mL. The bacterial suspension was cultured in BHIB supplemented with HFE and antibiotics, both individually and in combination. The concentrations of each antibacterial agent ranged from 1/8 × MIC to 4 × MIC. The experiment was carried out using the same method as mentioned for MIC determination. The results were analyzed and interpreted according to the fractional inhibitory concentration (FIC) index using the following formula: FIC index = (MIC of HFE in combination/MIC of HFE alone) + (MIC of antibiotic in combination/MIC of antibiotic alone). Synergy was defined as an FIC index of ≤0.5, indifference as an FIC index of >0.5 to ≤4, and antagonism as an FIC index of >4.

### 2.12. Statistical Analysis

Data were analyzed using one-way analysis of variance, followed by Tukey’s multiple comparison test. Statistically significant was defined as *p* < 0.05.

## 3. Results

### 3.1. Antibacterial Activity of the Herbal Formulation Ethanolic Extract (HFE) and Antibiotics Against S. pyogenes

The MICs of HFE and antibiotics against *S. pyogenes* are given in Table 1. The MIC and MBC of the HFE against *S. pyogenes* clinical strains were 8 µg/mL, whereas those against *S. pyogenes* ATCC 19615 were 16 µg/mL and 32 µg/mL, respectively. The MIC_90_ of ampicillin against *S. pyogenes* isolates was 2 µg/mL, with MIC and MBC ranges of 2–4 µg/mL. These values exceeded the CLSI susceptible breakpoint (≤0.25 µg/mL) [27]. The MIC_90_ of clindamycin and erythromycin against the tested bacteria was 0.03 µg/mL, which was below the susceptible MIC breakpoint (≤0.25 µg/mL). For vancomycin, MIC_50_ and MIC_90_ were 0.5 and 1 µg/mL, respectively. The results indicated that all streptococcal isolates were susceptible to vancomycin, clindamycin, and erythromycin.

### 3.2. Time-Kill Study of the Effect of the Herbal Formulation Ethanolic Extract on S. pyogenes

**Figure 1:**
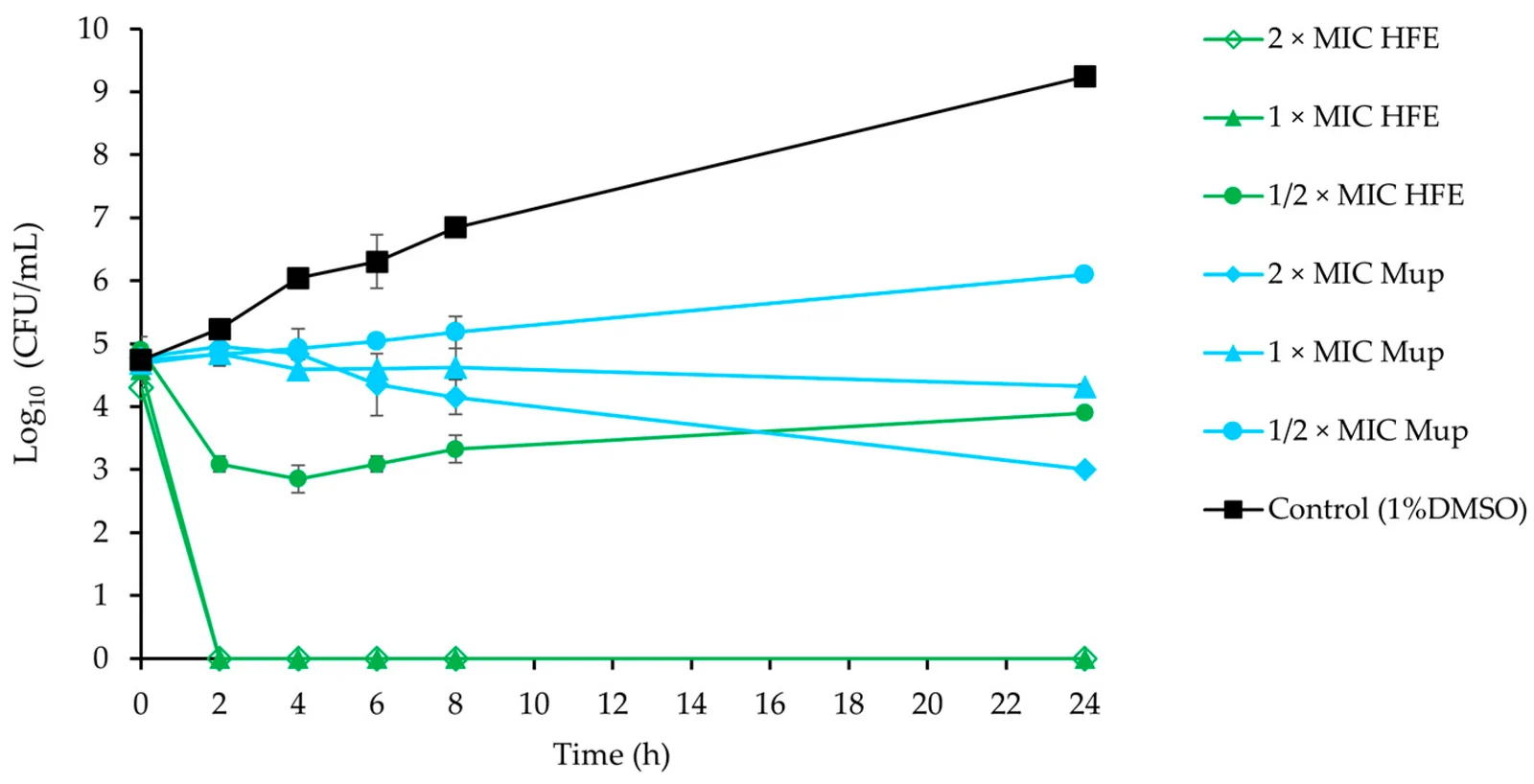
Time-kill curves for the effect of the herbal formulation ethanolic extract (HFE) and mupirocin at 1/2 × MIC, 1 × MIC, and 2 × MIC on *S. pyogenes* ATCC 19615. Bacterial cells treated with 1% DMSO served as the control. Data are presented as means, with range bars indicating the minimum and maximum values of the two replicates. Abbreviations: DMSO, dimethyl sulfoxide; HFE, herbal formulation ethanolic extract; Mup, mupirocin.

The antibacterial activity of HFE against *S. pyogenes* ATCC 19615, a representative strain, was further evaluated by the time-kill method. Mupirocin, a topical antibiotic used for bacterial skin infection treatment, was included. The number of viable bacterial cells after being treated with MIC (16 µg/mL) was reduced by 3 log folds (99.9%) within 2 h (Figure 1). Mupirocin at 2 × MIC could reduce the number of streptococcal cells by approximately 99% at 24 h of treatment. However, the results are due to the bacteriostatic activity of mupirocin. The results from both MIC and MBC values and the time-kill study indicated bactericidal activity of HFE against *S. pyogenes*.

### 3.3. Herbal Formulation Ethanolic Extract Induces Morphological Changes in S. pyogenes Cells

**Figure 2:**
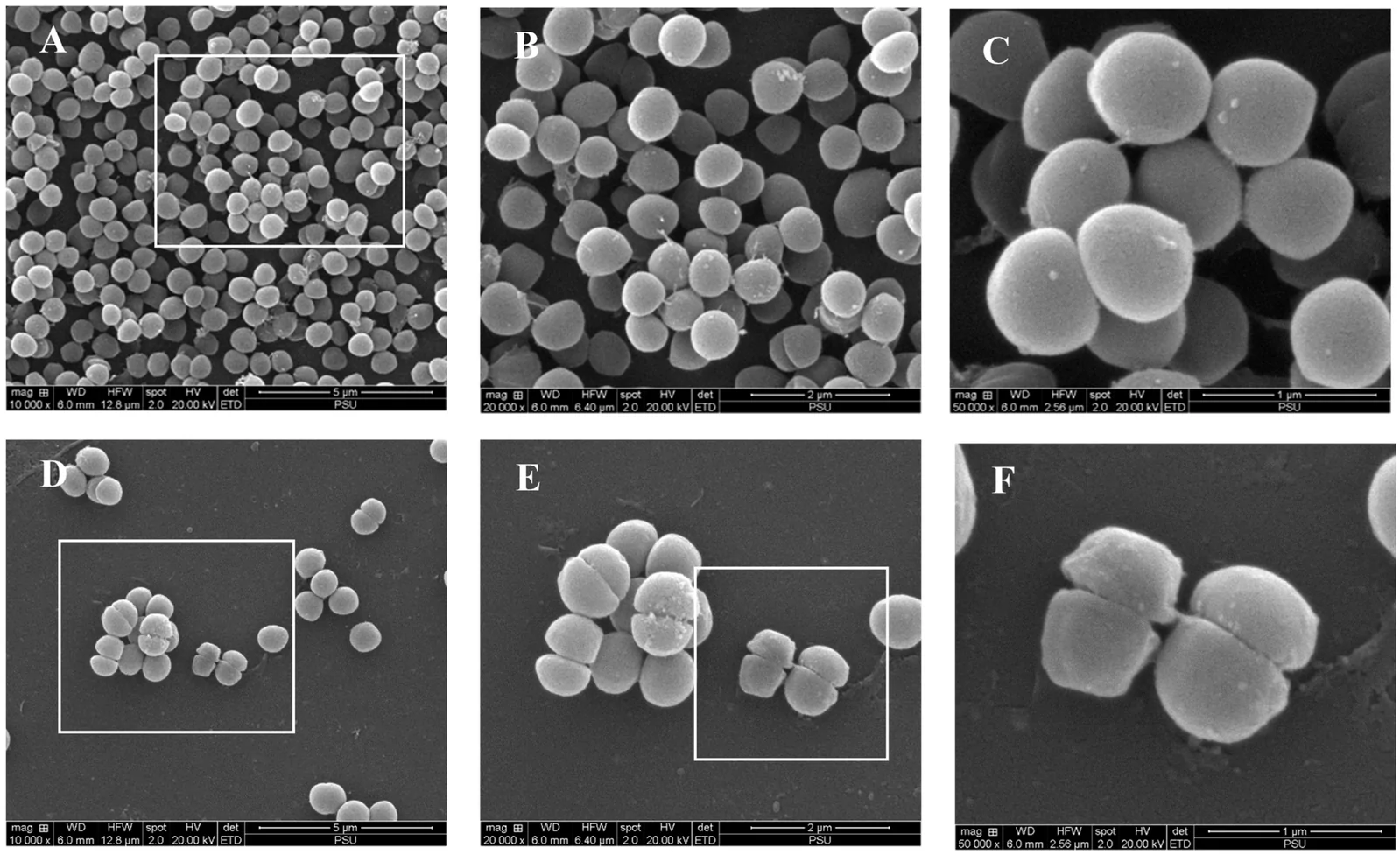
Scanning electron micrographs of untreated *S. pyogenes* ATCC 19615 cells (1% DMSO control; **A**–**C**) and cells treated with the herbal formulation ethanolic extract at 2 × MIC (**D**–**F**). Images were obtained at magnifications of 10,000× (**A**,**D**), 20,000× (**B**,**E**), and 50,000× (**C**,**F**).

**Figure 3:**
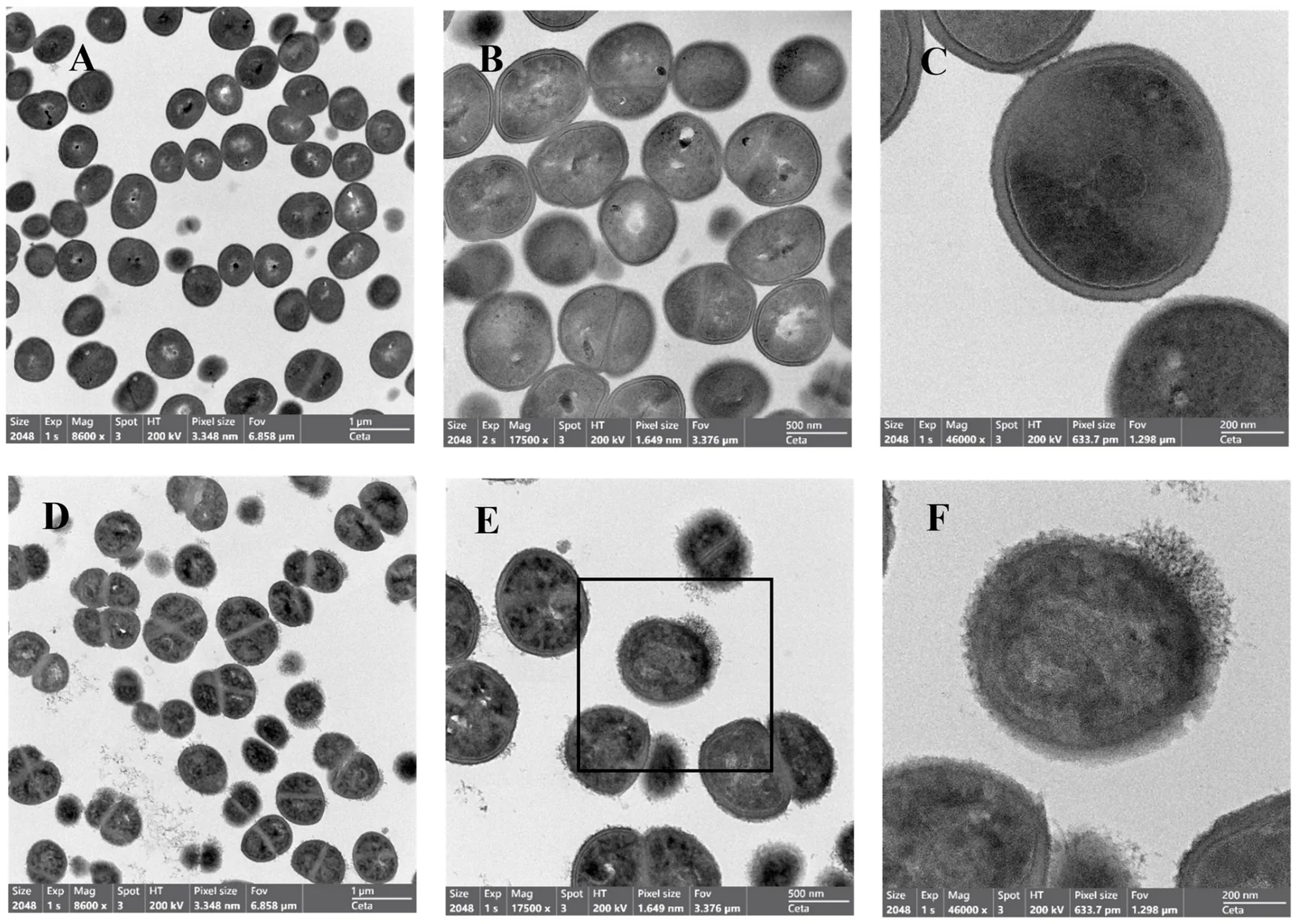
Transmission electron micrographs of untreated *S. pyogenes* ATCC 19615 cells (1% DMSO control; **A**–**C**) and cells treated with the herbal formulation ethanolic extract at 2 × MIC (**D**–**F**). Images were obtained at magnifications of 8600× (**A**,**D**), 17,500× (**B**,**E**), and 46,000× (**C**,**F**).

The morphology of *S. pyogenes* ATCC 19615 cells after exposure to 2 × MIC of the herbal formulation ethanolic extract was examined using SEM and TEM. SEM analysis showed that the streptococcal control cells treated with 1% DMSO exhibited a typical spherical (cocci) morphology with smooth surfaces (Figure 2A–C). In contrast, cells exposed to the herbal extract displayed noticeable surface deformation and shrinkage (Figure 2D–F). TEM micrographs further revealed numerous dense globular structures along the periphery of extract-treated cells (Figure 3D–F) compared with the 1% DMSO-treated control (Figure 3A–C). These morphological changes suggest that the herbal formulation ethanolic extract may affect the bacterial cell envelope.

### 3.4. Herbal Formulation Ethanolic Extract Reduces S. pyogenes Biofilm Formation

**Figure 4:**
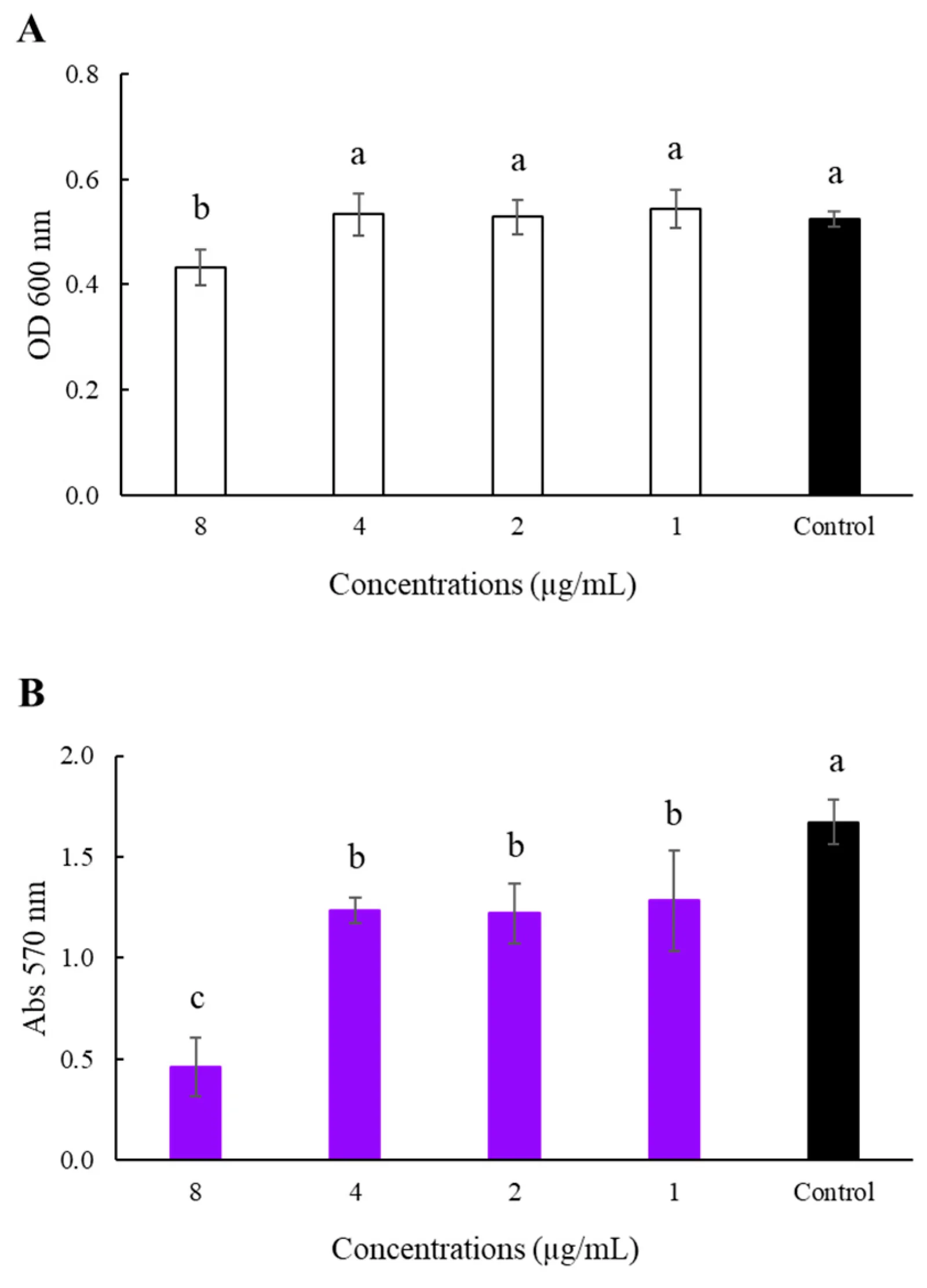
Biofilm formation of *Streptococcus pyogenes* ATCC 19615 in the presence of sub-inhibitory concentrations of the herbal formulation ethanolic extract compared with those treated with 1% DMSO as the control. The MIC of the herbal formulation extract was 16 µg/mL. (**A**) Bacterial growth measurement. (**B**) Biofilm formation determined by crystal violet staining assay. Values are presented as mean ± SD of triplicate experiments. Bars with different superscript letters, as determined by Tukey’s multiple comparison test, indicate statistical significance (*p* < 0.05).

**Figure 5:**
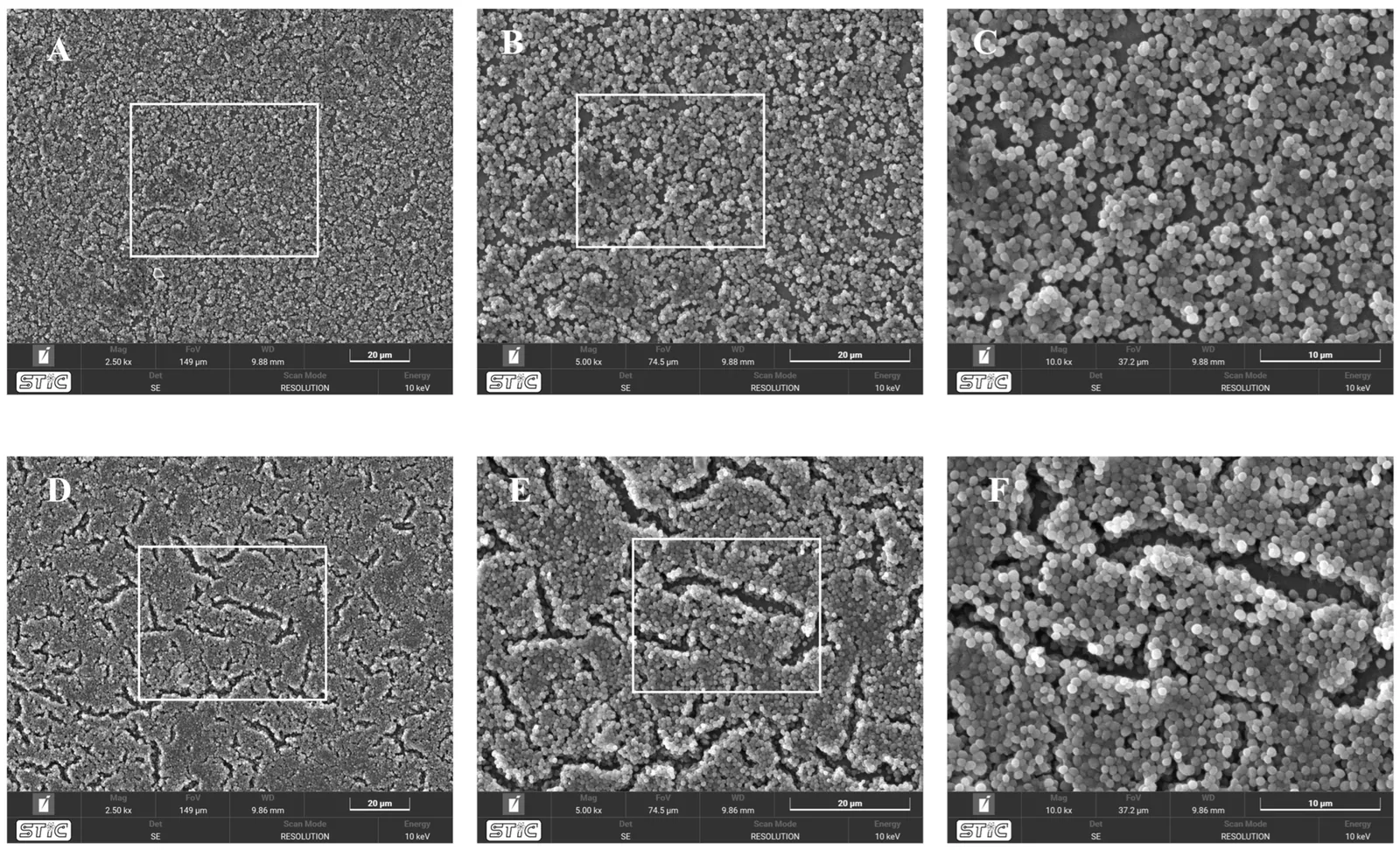
Scanning electron micrographs of *S. pyogenes* ATCC 19615 biofilm formation in the presence of 4 µg/mL (1/4 × MIC) of the herbal formulation ethanolic extract ((**A**) 2500×, (**B**) 5000×, and (**C**) 10,000×) compared with those treated with 1% DMSO as the control ((**D**) 2500×, (**E**) 5000×, and (**F**) 10,000×).

To confirm that the effect of HFE at sub-MICs on biofilm formation was not due to reduced bacterial growth, the growth of *S. pyogenes* was evaluated (Figure 4A). Treatment with HFE at 1/4, 1/8, and 1/16 × MIC did not significantly affect bacterial growth compared with the untreated control. However, treatment at 1/2 × MIC resulted in a lower OD600 value, indicating reduced bacterial growth. The effect of HFE on biofilm formation was investigated using the crystal violet assay. The results showed that HFE at sub-MICs inhibited *S. pyogenes* biofilm formation (Figure 4B). Treatment with HFE at 1/4, 1/8, and 1/16 × MIC reduced biofilm formation by approximately 30% compared with the control. A greater reduction was observed at 1/2 × MIC, with approximately 70% reduction in biofilm formation; however, reduced bacterial growth was also observed at this concentration (Figure 4B). Therefore, the observed reduction in biofilm formation at 1/2 × MIC may be partly attributable to reduced bacterial growth. SEM observations revealed that biofilm formation by *S. pyogenes* ATCC 19615 in the presence of 4 µg/mL (1/4 × MIC) of the herbal formulation ethanolic extract showed a reduction in bacterial cell density and surface coverage (Figure 5A–C) compared with the untreated control (Figure 5D–F).

### 3.5. Streptococcus pyogenes Established Biofilms Were Interfered by the Herbal Formulation Ethanolic Extract

**Figure 6:**
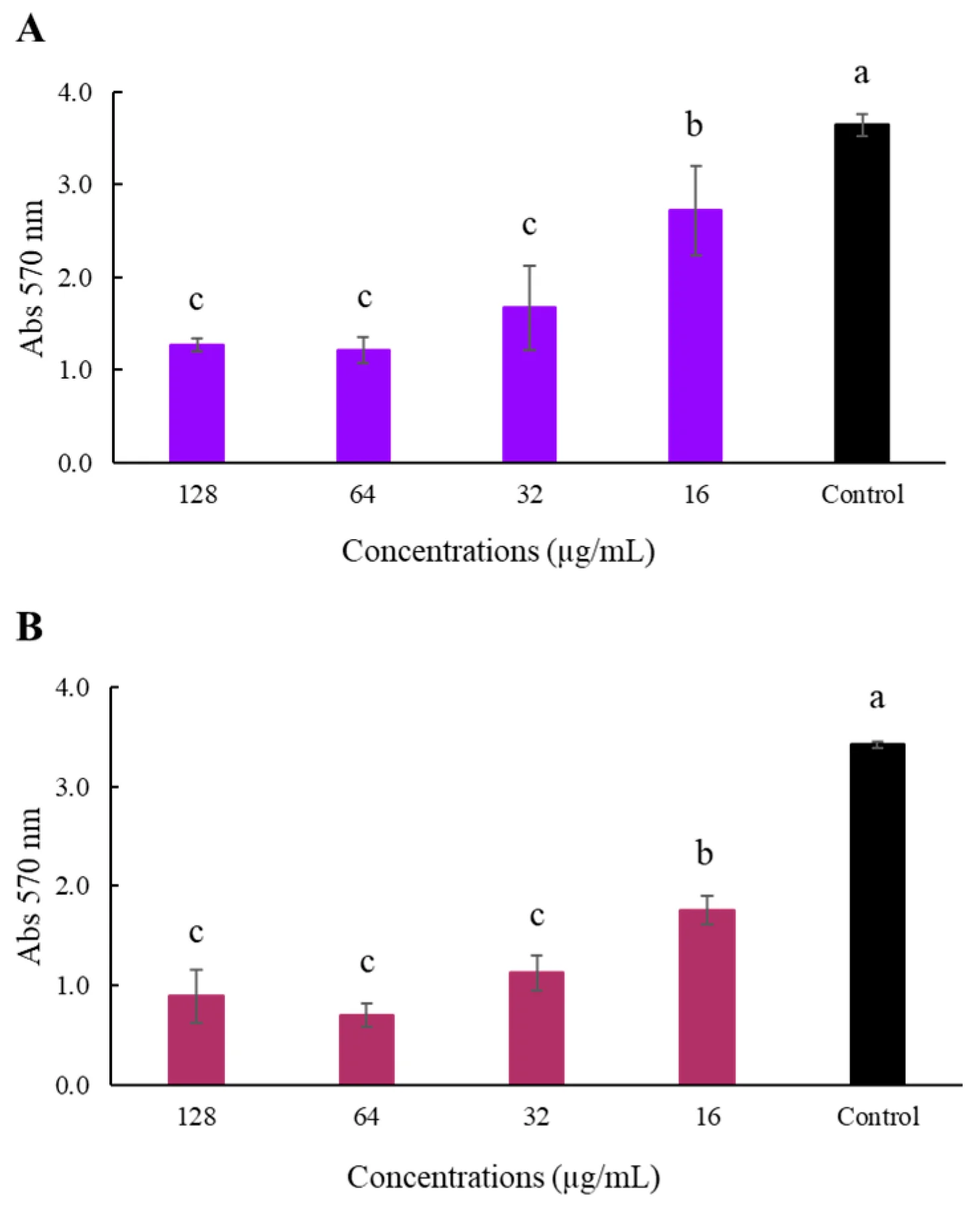
*S. pyogenes* ATCC 19615 established biofilms after treatment with supra-MICs of the herbal formulation ethanolic extract compared with those treated with 1% DMSO as the control. The MIC of the herbal formulation ethanolic extract was 16 µg/mL. (**A**) Total biofilm biomass of the established biofilms determined by the crystal violet staining assay. (**B**) Bacterial viability in the established biofilms determined by the MTT assay. Bars with different superscript letters, as determined by Tukey’s multiple comparison test, indicate statistical significance (*p* < 0.05).

**Figure 7:**
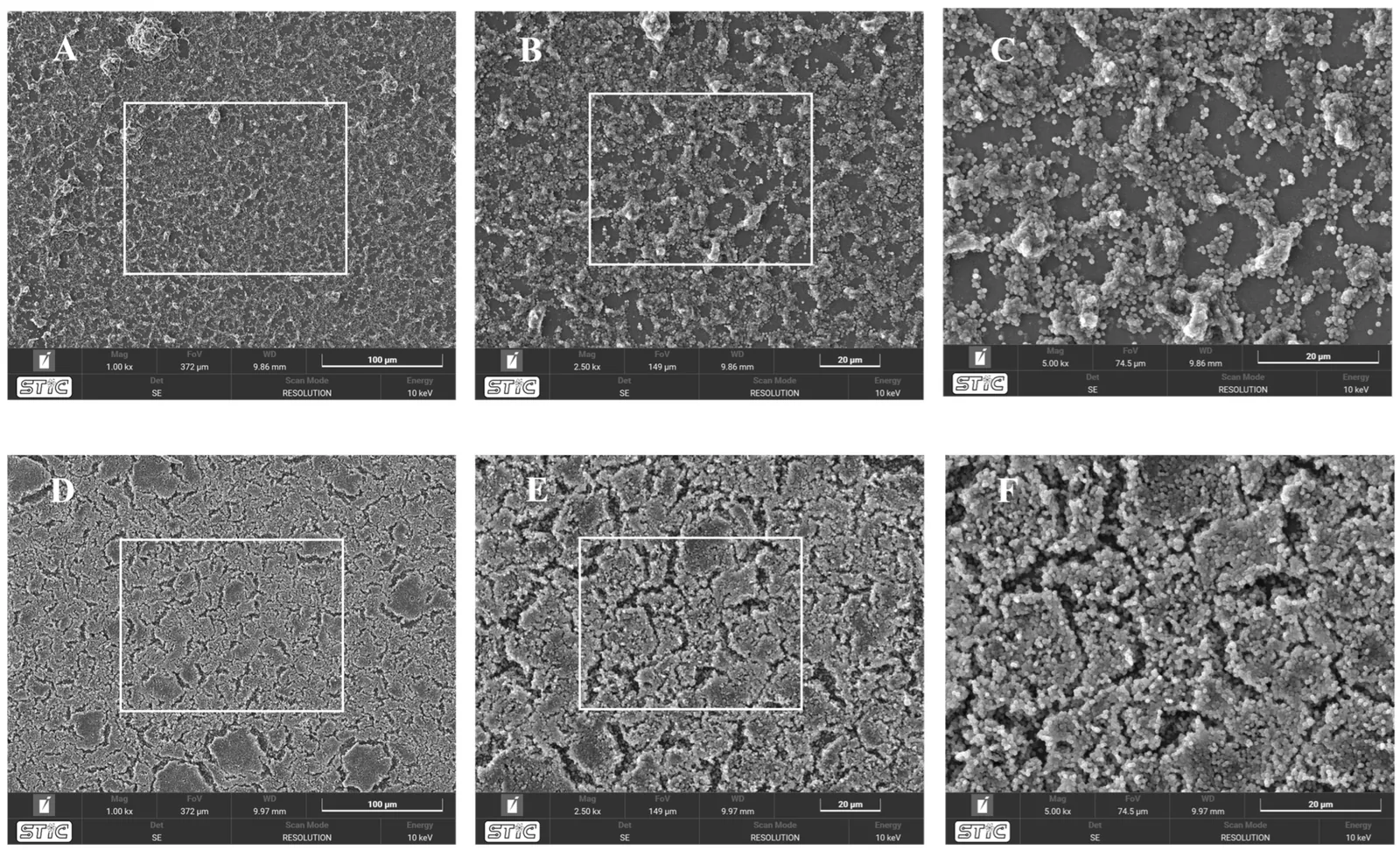
Scanning electron micrographs of established biofilms of *Streptococcus pyogenes* ATCC 19615 after treatment with 32 µg/mL (2 × MIC) of the herbal formulation ethanolic extract ((**A**) 1000×, (**B**) 2500×, and (**C**) 5000×) compared with those treated with 1% DMSO as the control ((**D**) 1000×, (**E**) 2500×, and (**F**) 5000×).

The established biofilms, via the crystal violet assay, revealed a reduction in biomass after treatment with HFE (1–8 × MIC) in a dose-dependent manner. At 2 × MIC (32 µg/mL) of the extract, the total biofilm biomass was reduced by about 50% when compared to the untreated control (Figure 6A). Similarly, biofilm cell viability, via the MTT assay, decreased in a dose-dependent trend in the presence of the ethanolic extract compared to the control (Figure 6B). Scanning electron micrographs showed a marked reduction in streptococcal cell layers and the presence of scattered cell clusters following treatment with 32 µg/mL (2 × MIC) of HFE (Figure 7A–C). In contrast, untreated control biofilms exhibited a dense biofilm structure with thick layers of bacterial cells (Figure 7D–F).

### 3.6. Combination Effects of the Herbal Formulation Ethanolic Extract and Antibiotics

**Figure 8:**
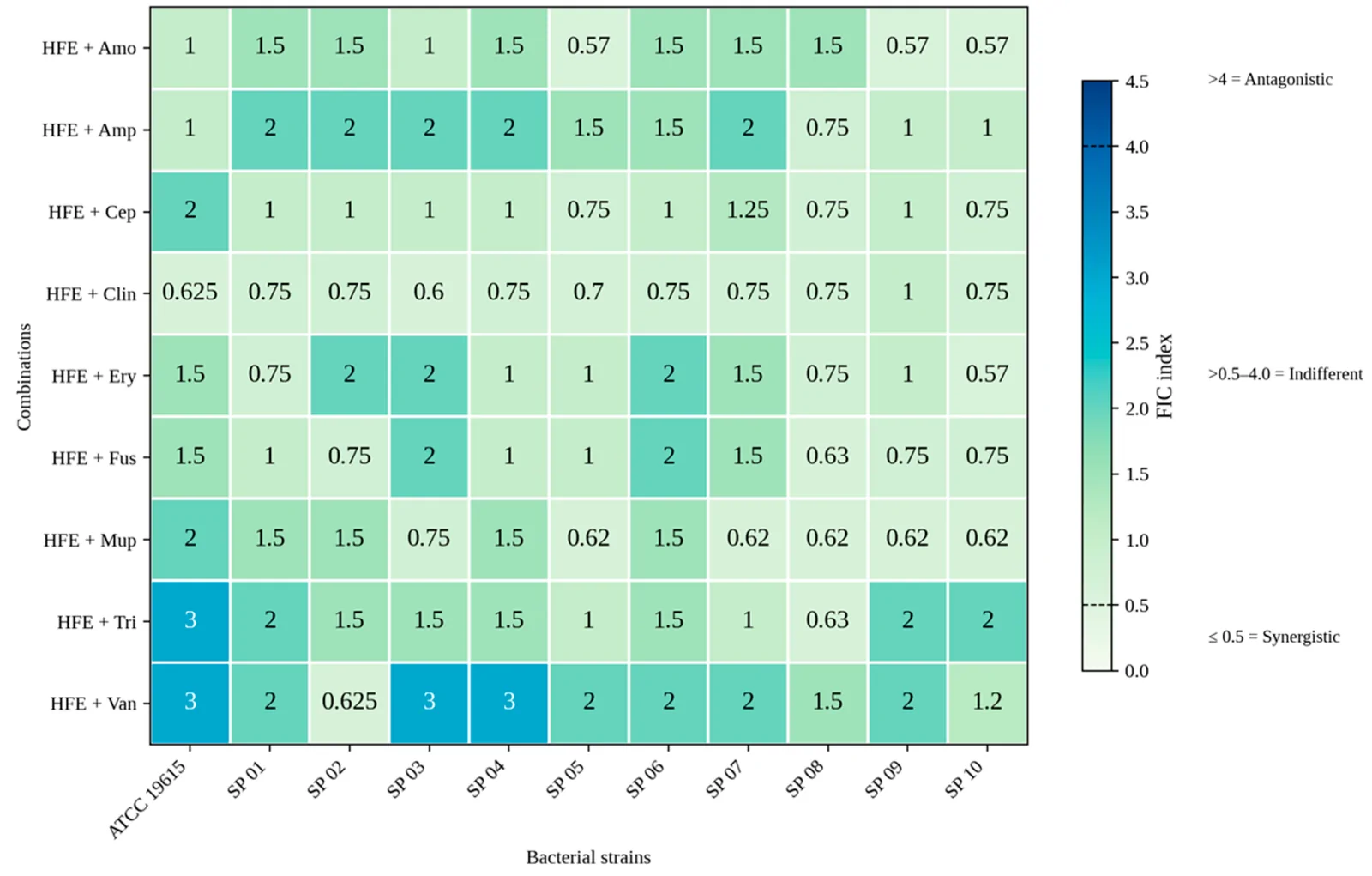
Fractional inhibitory concentration (FIC) indices for combinations of the herbal formulation ethanolic extract (HFE) and antibiotics against *S. pyogenes*. Abbreviations: Amo, amoxicillin; Amp, ampicillin; Cep, cephalexin; Clin, clindamycin; Ery, erythromycin; Fus, fusidic acid; Mup, mupirocin; Tri, trimethoprim; Van, vancomycin.

The microdilution checkerboard assay was employed to evaluate the interaction between HFE and antibiotics. Combinations of HFE with antibiotics—including amoxicillin, ampicillin, cephalexin, clindamycin, erythromycin, fusidic acid, mupirocin, trimethoprim, and vancomycin—exhibited FIC indexes ranging from 0.57 to 3.00, indicating an indifferent interaction of all combinations against all tested bacterial strains (Table 2 and Figure 8). No antagonistic effect was observed in the combination of HFE with all tested antibiotics.

## 4. Discussion

*S. pyogenes* is an important pathogen that can cause infections in children and elderly populations, ranging from non-invasive conditions, including impetigo and sore throats, to severe invasive infections, such as necrotizing fasciitis [31]. It remains a significant global cause of morbidity and mortality, particularly in regions with limited resources [4]. Furthermore, the antibiotic resistance of *S. pyogenes* has become a problem for infection management. In the present study, the MIC_90_ of ampicillin against the tested *S. pyogenes* isolates exceeded the CLSI susceptibility breakpoint, indicating reduced susceptibility to ampicillin. Similar observations have been reported in recent studies, suggesting that isolates with decreased susceptibility to ampicillin may emerge [11,32]. However, 100% ampicillin susceptibility has also been reported in *S. pyogenes* isolates [33,34]. Although resistance to vancomycin, erythromycin, and clindamycin was not detected in this study, resistance to these antibiotics has been reported from various global regions [11,32,33,34,35].

Due to the continuous increase in antibiotic resistance, alternative approaches for the treatment of streptococcal infections are urgently needed. Herbal formulations are a promising source of antibacterial agents. Antistreptococcal activities of medicinal plant formulations have been reported. The ethanolic extracts of four polyherbal formulations in Southern Thailand exhibited antibacterial activity against *S. pyogenes* [17]. In addition, the ethyl acetate and methanolic extracts from medicinal plants used in Thai longevity formulations possessed antibacterial activity against *S. pyogenes* with MIC values ranging from 19.5 to 1250 µg/mL [19]. Mahanintangtong, a traditional polyherbal formulation listed in Thailand’s National List of Essential Medicines and consisting of several medicinal plant ingredients, also exhibited antibacterial activity against *S. pyogenes*, with an MIC of 0.078 mg/mL, whereas the MBC was greater than 5 mg/mL [18]. Our results showed promising antibacterial activity of the herbal formulation ethanolic extract with a MIC_90_ of 8 µg/mL, when compared with previous reports on the antibacterial activity of polyherbal formulations against *S. pyogenes*. Notably, all clinical isolates showed lower MIC and MBC values (8 µg/mL) than the reference strain *S. pyogenes* ATCC 19615 (16 and 32 µg/mL, respectively). These differences may reflect strain-specific characteristics that influence susceptibility to HFE. However, the underlying mechanisms responsible for the higher susceptibility of the clinical isolates were not investigated in the present study. Moreover, the ethanolic extract possessed bactericidal activity against *S. pyogenes*, as indicated by the MIC and MBC values and the time-kill curves showing a 99.9% reduction in bacterial cells within 2 h in the presence of the extract at the MIC. These findings were further supported by the morphological changes observed in treated cells. Electron micrographs revealed irregular protrusions on the cell surface of treated cells, whereas untreated cells exhibited relatively smooth surfaces. Together, these observations suggest that the herbal formulation ethanolic extract may affect the bacterial cell wall, which is essential for maintaining cell integrity, shape, and viability. Disruption of the cell wall may compromise bacterial viability and lead to cell death. Peptidoglycan, a major component of the bacterial cell wall, is the primary target of cell wall synthesis inhibitors. Although the exact mechanism of the extract remains unclear, the observed morphological changes resemble those caused by cell wall synthesis inhibitors. These antibiotics inhibit different steps of peptidoglycan synthesis, leading to impaired cell wall formation [36,37,38]. The time-kill assay was performed in duplicate, which is a limitation of the present study. Further studies using biological replicates and diverse clinical isolates are needed to confirm the bactericidal activity of HFE.

A biofilm is a sessile community of microorganisms enclosed in an extracellular polymeric matrix and attached to a surface or to each other [39]. Biofilm is an important virulence factor of *S. pyogenes* that contributes to the persistence of infections. Biofilm-producing *S. pyogenes* strains from various sources are genetically diverse, resulting in variations in biofilm phenotypes among strains [6]. Previous reviews have highlighted the important role of biofilms in *S. pyogenes* infections, antibiotic resistance, and antimicrobial therapeutics [40]. Because bacterial biofilms are more resistant to antibiotic treatment, effective antibiofilm agents are needed to control biofilm-associated infections. Our findings showed that the herbal formulation ethanolic extract affected both *S. pyogenes* biofilm formation and established biofilms. HFE at sub-MICs inhibited *S. pyogenes* biofilm formation. However, reduced bacterial growth was also observed at 1/2 × MIC, suggesting that the reduction in biofilm formation at this concentration may be partly associated with reduced bacterial growth. Furthermore, the ethanolic extract also reduced cell viability and disrupted established biofilms, as confirmed by fewer bacterial layers and cell clusters than those observed in the untreated control. Several previous studies have reported the antibiofilm activity of medicinal plants against *S. pyogenes* by inhibiting different stages of biofilm formation [41,42,43,44]. In this study, the herbal formulation ethanolic extract interfered with both biofilm formation and established biofilms depending on the concentrations at sub-MIC and supra-MIC levels, respectively. The herbal formulation ethanolic extract contains a variety of phytochemical constituents, and its mechanisms of action may involve several targets in *S. pyogenes* cells rather than a single target or pathway. A limitation of this study is that the phytochemical composition of HFE was not characterized, and the specific compounds contributing to its antibacterial and antibiofilm activities could not be determined.

The combination of herbal extracts with antibiotics has been investigated as a potential approach for improving antibacterial activity. The combination of antibacterial agents may result in synergy, indifference, or antagonism. Previous studies have demonstrated that the combination of conventional antibiotics and natural products is a potential strategy for enhancing antibacterial activity through synergistic effects [9,20,45,46]. However, our findings showed indifferent interactions between the herbal formulation ethanolic extract and the tested antibiotics, including amoxicillin, ampicillin, cephalexin, clindamycin, erythromycin, fusidic acid, mupirocin, trimethoprim, and vancomycin. Although the FICI values varied among the tested antibiotics, ranging from 0.57 to 3.00, all combinations were classified as indifferent according to the criteria used in this study, with none meeting the criteria for synergy or antagonism. Previous studies have also reported indifferent interactions between the ethanolic extracts of *Salvia officinalis* or *Plectranthus ornatus* and chloramphenicol against *S. aureus* [47]. The combination of the ethanolic extract of *Ziziphus mucronata* and antibiotics against clinically important bacteria resulted in synergistic (54.17%), additive (27.78%), indifferent (16.67%), and antagonistic (1.39%) interactions, depending on the bacterial strains [48]. Herbal medicines in combination with antibiotics have been reported to exhibit positive, negative, and non-interactive (indifferent) interactions depending on the bacterial species [49]. The interaction between herbal extracts and antibiotics may vary depending on the phytochemical composition, the antibiotic used, and the bacterial species. Although HFE did not show synergistic interactions with the tested antibiotics, the absence of antagonistic interactions indicates that HFE did not interfere with the antibacterial activity of the tested antibiotics under the conditions evaluated. However, this finding does not establish a therapeutic or complementary benefit of combining HFE with antibiotics. Further studies are needed to determine the clinical relevance of these combinations.

## 5. Conclusions

The ethanolic extract of the herbal formulation containing *Anacardium occidentale* pericarps, *Zingiber montanum* rhizomes, and *Aloe vera* dried leaf latex exhibited bactericidal activity against *S. pyogenes*. In addition, it inhibited biofilm formation and disrupted established biofilms. The FIC indices indicated indifferent interactions when the extract was combined with the tested antibiotics. These findings highlight the potential of HFE for further investigation against *S. pyogenes*. Additional studies are needed to evaluate its safety and efficacy in appropriate in vivo models before clinical application.

## Figures and Tables

**Table 1:** Antibacterial activity of the herbal formulation ethanolic extract (HFE) and antibiotics against *Streptococcus pyogenes*.

Antibacterial Agents	*S. pyogenes* Strains (*n* = 10)				*S. pyogenes* ATCC 19615		MIC Breakpoints (µg/mL)	
	MIC_50_ (µg/mL)	MIC_90_ (µg/mL)	MIC Ranges (µg/mL)	MBC Ranges (µg/mL)	MIC (µg/mL)	MBC (µg/mL)	Susceptible	Resistant
HFE	8	8	8	8	16	32	-	-
Amoxicillin	0.015	0.03	0.015–0.03	0.015–0.03	0.25	0.25	-	-
Ampicillin	2	2	2–4	2–4	2	2	≤0.25	-
Cephalexin	1	1	0.5–1	0.5–1	1	1	-	-
Clindamycin	0.03	0.03	0.015–0.03	0.03–0.125	0.25	0.25	≤0.25	≥1
Erythromycin	0.03	0.03	0.015–0.06	0.06	0.5	>4	≤0.25	≥1
Fusidic acid	4	8	4–8	4->8	0.25	0.25	-	-
Mupirocin	0.125	0.125	0.125–0.25	0.125–0.25	0.125	0.125	-	-
Trimethoprim	32	>32	1->32	4->32	1	1	-	-
Vancomycin	0.5	1	0.5–1	0.5–1	2	2	≤1	-

Ampicillin, clindamycin, erythromycin, and vancomycin: interpretation according to CLSI MIC breakpoints. Amoxicillin, cephalexin, fusidic acid, mupirocin, and trimethoprim: MIC breakpoints are not mentioned in CLSI.

**Table 2:** Interactions of the herbal formulation ethanolic extract and antibiotics, together against *S. pyogenes*.

*S. pyogenes* Strains	Antibacterial Combinations	HFE (µg/mL)		Antibiotics (µg/mL)		FICI	Interpretation
		MIC Combined	MIC Alone	MIC Combined	MIC Alone		
ATCC 19615	HFE + Amo	16	32	0.125	0.25	1.00	indifference
	HFE + Amp	16	32	1	2	1.00	indifference
	HFE + Cep	32	32	2	2	2.00	indifference
	HFE + Clin	4	32	0.125	0.25	0.625	indifference
	HFE + Ery	32	64	0.5	0.5	1.50	indifference
	HFE + Fus	32	64	0.25	0.25	1.50	indifference
	HFE + Mup	64	64	0.125	0.125	2.00	indifference
	HFE + Tri	64	64	1	0.5	3.00	indifference
	HFE + Van	64	64	2	1	3.00	indifference
SP 01	HFE + Amo	4	8	0.015	0.015	1.50	indifference
	HFE + Amp	8	8	1	1	2.00	indifference
	HFE + Cep	4	8	0.25	0.5	1.00	indifference
	HFE + Clin	4	8	0.0075	0.03	0.75	indifference
	HFE + Ery	2	8	0.015	0.03	0.75	indifference
	HFE + Fus	4	8	2	4	1.00	indifference
	HFE + Mup	4	8	0.125	0.125	1.50	indifference
	HFE + Tri	8	8	32	32	2.00	indifference
	HFE + Van	8	8	0.5	0.5	2.00	indifference
SP 02	HFE + Amo	4	8	0.015	0.015	1.50	indifference
	HFE + Amp	4	4	1	1	2.00	indifference
	HFE + Cep	4	8	0.5	1	1.00	indifference
	HFE + Clin	4	8	0.0075	0.03	0.75	indifference
	HFE + Ery	8	8	0.03	0.03	2.00	indifference
	HFE + Fus	4	8	1	4	0.75	indifference
	HFE + Mup	4	8	0.125	0.125	1.50	indifference
	HFE + Tri	4	8	16	16	1.50	indifference
	HFE + Van	4	8	0.06	0.5	0.625	indifference
SP 03	HFE + Amo	4	8	0.0075	0.015	1.00	indifference
	HFE + Amp	4	4	1	1	2.00	indifference
	HFE + Cep	4	8	0.25	0.5	1.00	indifference
	HFE + Clin	4	8	0.003	0.03	0.60	indifference
	HFE + Ery	8	8	0.03	0.03	2.00	indifference
	HFE + Fus	8	8	4	4	2.00	indifference
	HFE + Mup	2	8	0.0625	0.125	0.75	indifference
	HFE + Tri	4	8	32	32	1.50	indifference
	HFE + Van	8	8	1	0.5	3.00	indifference
SP 04	HFE + Amo	4	8	0.015	0.015	1.50	indifference
	HFE + Amp	4	4	1	1	2.00	indifference
	HFE + Cep	4	8	0.5	1	1.00	indifference
	HFE + Clin	4	8	0.0075	0.03	0.75	indifference
	HFE + Ery	4	8	0.015	0.03	1.00	indifference
	HFE + Fus	4	8	2	4	1.00	indifference
	HFE + Mup	4	8	0.125	0.125	1.50	indifference
	HFE + Tri	4	8	32	32	1.50	indifference
	HFE + Van	8	8	1	0.5	3.00	indifference
SP 05	HFE + Amo	4	8	0.001	0.015	0.57	indifference
	HFE + Amp	4	8	1	1	1.50	indifference
	HFE + Cep	4	8	0.125	0.5	0.75	indifference
	HFE + Clin	4	8	0.003	0.015	0.70	indifference
	HFE + Ery	4	8	0.0075	0.015	1.00	indifference
	HFE + Fus	4	8	2	4	1.00	indifference
	HFE + Mup	4	8	0.015	0.125	0.62	indifference
	HFE + Tri	4	8	1	2	1.00	indifference
	HFE + Van	8	8	0.5	0.5	2.00	indifference
SP 06	HFE + Amo	4	8	0.015	0.015	1.50	indifference
	HFE + Amp	4	8	1	1	1.50	indifference
	HFE + Cep	4	8	0.25	0.5	1.00	indifference
	HFE + Clin	4	8	0.0075	0.03	0.75	indifference
	HFE + Ery	8	8	0.03	0.03	2.00	indifference
	HFE + Fus	8	8	4	4	2.00	indifference
	HFE + Mup	4	8	0.06	0.06	1.50	indifference
	HFE + Tri	4	8	32	32	1.50	indifference
	HFE + Van	8	8	1	1	2.00	indifference
SP 07	HFE + Amo	4	8	0.03	0.03	1.50	indifference
	HFE + Amp	8	8	2	2	2.00	indifference
	HFE + Cep	2	8	0.5	0.5	1.25	indifference
	HFE + Clin	4	8	0.0075	0.03	0.75	indifference
	HFE + Ery	4	8	0.015	0.015	1.50	indifference
	HFE + Fus	8	8	2	4	1.50	indifference
	HFE + Mup	4	8	0.015	0.125	0.62	indifference
	HFE + Tri	4	8	32	64	1.00	indifference
	HFE + Van	8	8	1	1	2.00	indifference
SP 08	HFE + Amo	4	8	0.03	0.03	1.50	indifference
	HFE + Amp	4	8	0.5	2	0.75	indifference
	HFE + Cep	4	8	0.125	0.5	0.75	indifference
	HFE + Clin	4	8	0.0037	0.015	0.75	indifference
	HFE + Ery	4	8	0.0075	0.03	0.75	indifference
	HFE + Fus	4	8	0.5	4	0.63	indifference
	HFE + Mup	4	8	0.015	0.125	0.62	indifference
	HFE + Tri	4	8	1	8	0.63	indifference
	HFE + Van	4	8	0.5	0.5	1.50	indifference
SP 09	HFE + Amo	4	8	0.001	0.015	0.57	indifference
	HFE + Amp	4	8	1	2	1.00	indifference
	HFE + Cep	4	8	0.25	0.5	1.00	indifference
	HFE + Clin	4	8	0.0075	0.015	1.00	indifference
	HFE + Ery	4	8	0.0075	0.015	1.00	indifference
	HFE + Fus	4	8	1	4	0.75	indifference
	HFE + Mup	4	8	0.015	0.125	0.62	indifference
	HFE + Tri	4	4	1	1	2.00	indifference
	HFE + Van	8	8	0.5	0.5	2.00	indifference
SP 10	HFE + Amo	4	8	0.001	0.015	0.57	indifference
	HFE + Amp	4	8	1	2	1.00	indifference
	HFE + Cep	4	8	0.125	0.5	0.75	indifference
	HFE + Clin	4	8	0.0075	0.03	0.75	indifference
	HFE + Ery	4	8	0.001	0.015	0.57	indifference
	HFE + Fus	4	8	1	4	0.75	indifference
	HFE + Mup	4	8	0.015	0.125	0.62	indifference
	HFE + Tri	4	4	1	1	2.00	indifference
	HFE + Van	2	8	0.5	0.5	1.25	indifference

## Data Availability

The original contributions presented in this study are included in the article. Further inquiries can be directed to the corresponding authors.
